# Healthcare service utilization of hill tribe children in underserved communities in thailand: Barriers to access

**DOI:** 10.1186/s12913-022-08494-1

**Published:** 2022-09-02

**Authors:** Katemanee Moonpanane, Khanittha Pitchalard, Jintana Thepsaw, Onnalin Singkhorn, Chomnard Potjanamart

**Affiliations:** School of Nursing, Mae Fah Luang University, Chiang Rai, Thailand

**Keywords:** Hill tribe children, Healthcare utilization, Health inequality, Thailand

## Abstract

**Background:**

Hill tribe children, an ethnic minority group in Thailand, experience wide-ranging social and health inequalities. Previous reports indicate that hill tribe children, especially age under 5 years, face social health disadvantages but little is known about the underlying causes. Exploring healthcare utilization among hill tribe children is therefore essential and it may well provide some insight.

**Methods:**

A qualitative study was conducted using purposive sampling techniques to recruit participants based on our criteria. In-depth interviews and focus-group discussions were employed to explore the experiences of parents (*n* = 20), community leaders (*n* = 20), and healthcare providers (*n* = 20) when caring for children aged under 5 years. Interview transcripts were coded, and thematic analysis was then performed.

**Results:**

The participants shared their experiences with accessing healthcare services in underserved areas. *Barriers to access* was the central theme identified. Sub-themes included: (1) distance matters, (2) education and socioeconomic deprivation, (3) lack of cultural sensitivity, (4) communication problems, (5) tradition, beliefs, and differences in cultural practice, (6) lack of child health professionals, and (7) bureaucratic hurdles.

**Conclusions:**

Healthcare services and environments must be transformed to provide healthcare services, education, and information appropriate to the cultures and beliefs prevalent in the hill tribe population.

**Supplementary Information:**

The online version contains supplementary material available at 10.1186/s12913-022-08494-1.

## Introduction

Health equity is a key component of the 2030 Sustainable Development Goals (SDGs) developed by the United Nations (UN), which seeks to “leave no one behind” in the achieving the target of equal access to healthcare [1, 2]. This requires the continuous and vigilant provision of healthcare services and interventions that are appropriate for the communities receiving them [3]. Members of the UN have demonstrated a growing commitment to addressing the issue of health inequality [4]. The Thai government has become concerned with this issue and is committed to reducing health inequality by designing and implementing a universal healthcare coverage scheme (UC) for all Thai citizens who have ID card [5–7]. However, this scheme has many well-documented problems, including difficulties with the process of obtaining referrals to alternative hospitals, inadequate numbers of healthcare professionals.

Apidechkul et al. [8] reported that more than 30% or 540,000 people who do not have Thai identification cards (ID cards) which are used to gain access to free public services including seeking employment, education for children, and healthcare services. Therefore, the absence of a Thai ID card leads to an inability to fully access healthcare services that support a good quality of life [9, 10]. Furthermore, those who lack a Thai ID card face stigma from people around them through several processes. The severity of such stigma and discrimination are barriers to individuals’ health and well-being. Meanwhile, inequities in healthcare access can be found regionally, especially among regions with ethnic minorities such as hill tribe groups [11, 12].

In Thailand, the hill tribes are a prime example of disparities in healthcare. Hill tribe people are considered “minority people” or “alien” and thus separate from the general population [13]. They have fewer opportunities to reap the benefits of healthcare services and are more likely to receive inappropriate treatment [14, 15]. Moreover, hill tribe populations face significant obstacles in achieving optimal health due to geographical isolation and poverty [11, 14, 16]. Several previous studies in Thailand have also found that hill tribe children have poor health conditions. For example, 30.0% of children under 1 years old received Measle Mumps and Rubella (MMR) vaccine, while 70.0 percent received the 3^rd^ dose of the diphtheria, tetanus, pertussis, and polio vaccine (DTP3/OPV3) [8], compared to Thai children in general, who have received > 90% of the Expanded Program on Immunization (EPI) since the year 2000. As a result of the low EPI rate, in 2020, 6.71 hill tribe children per 100,000 population were reported to have measles infection with 24.8% of these children being under 5 years of age [17, 18]. This is perhaps due to the many barriers hill tribe parents face in accessing in healthcare services for their children [18, 19]. Not only is the risk for infectious disease high, but malnutrition is also a significant problem among hill tribe children. Overall, 38% of hill tribe children under 5 years of age demonstrated stunted growth, which is much higher than the stunting rate for other groups of Thai children, at 10–15% [20–22].

Chiang Rai Province has the largest hill tribe population, with 30% of the 4 million hill tribe people in Thailand. Most hill tribes people live at high elevations along the border of Thailand, Myanmar, and Lao PDR. These people are classified into six main groups: Akha, Lahu, Hmong, Yao, Karen, and Lisu. Each has its own unique cultures, customs, beliefs, traditions, and languages. Almost all hill tribes live in mountainous areas, work in the agricultural sector, and live below the poverty line which affects their health condition and healthcare utilization. [8, 15, 16].

There is a lack of research on the healthcare utilization of hill tribe populations in Thailand. Most previous studies have focused on the prevalence or incidence of infectious diseases, nutritional status, or factors related to vaccination in children and adolescent hill tribe members [13, 18–25]. This current study is the first qualitative research aimed at evaluating healthcare service utilization for these hill tribe children based on the experiences of parents, healthcare providers, and community leaders. We focus on these three groups’ perceptions, culture, and lifestyles with a special emphasis on access to healthcare. The findings will help to improve hill tribe children’s access to healthcare that is appropriate to their health, age, development, culture and beliefs. The findings will also inform future empirical clinical and community interventions and policies.

## Materials and methods

Qualitative methods, in-depth interviews, and focus group discussions (FGDs) [26] were employed to explore healthcare service delivery and reasons for the underutilization of healthcare services for hill tribe children age under 5 years in Chiang Rai Province, Thailand.

### Settings and participants

Thoeng and Mae Fah Luang districts, were selected as the areas of focus for our study based on their population’s low economic status, their remoteness from the provincial facilities, and their large hill tribe populations. In these districts, there is one medium-sized district hospital that serves a population of 3,000–8,000 persons [27]. These district hospitals work closely with a network of 10–14 sub-district hospitals.

Participants included key community leaders, healthcare providers, and hill tribe parents from these two districts. Interviewees with multiple backgrounds were included to increase the validity of the findings and gain a thorough understanding of healthcare service utilization in hill tribe children. The participants were purposively selected by a nurse practitioner in a primary care setting in these regions. Eligible participants included 1) hill tribe parents who had more than 2 years of experience in caring for children aged under 5 years and fluent in Thai 2) healthcare providers who had more than 5 years’ experience in healthcare services and delivery for hill tribe children aged under 5 years in these areas, and 3) community leaders who had facilitated the use of essential healthcare services for children (0–5 years) in their community and were fluent in Thai. These participants were chosen because they would provide valuable insights into child health and healthcare utilization and were willing to participate in the study. The study protocol was approved by the Human Ethics Research Committee, Chiang Rai Public Health Provincial Office (CRPPHO 51/2562).

### Data collection

Data were collected between September 2020 and August 2021 in two disadvantaged districts in Chiang Rai province. The primary investigator set up the three-day research workshop for team members to mentor and train junior qualitative researchers in a way that encourages critical and reflexive engagement with the data. A total of 60 in-depth interviews were conducted by the research team members. This interview guide was developed based on initial consultations with the integrated project partners to gather information (Supplement 1). FGDs were conducted to obtain the perceptions of participants (6–10 participants per time) regarding their experiences accessing healthcare services and facilitators and barriers to healthcare access. The Principal Investigator (KP) and co-author (KM) engaged in five FGDs with key informants in each group. FGDs were considered relevant to grasp the perceptions, attitudes, beliefs, and experiences of the participants who had the same background, trust each other, and to encourage the expression of personal views in a natural setting. The group discussion focused on topics that emerged from in-depth interviews, relevant information, potential barriers, and possible suggestions for improvement [26, 28]. The interviews and FGDs were arranged at the participants’ convenience, they were mostly held at participants’ homes as well as at the community meeting hall range from 45–70 min. As participants narrated their stories, probing questions were asked to assist the participants to explain the situation. FDGs and Interviews with the all participants ranged from 65–100 min and all interviews were audio-recorded and subsequently transcribed with the participants’ permission.

### Data analysis

Data were analyzed using thematic analysis. The thematic analysis method is useful for analyzing rich, detailed, and complex text – in this case, transcript—data. It is also particularly practical when analyzing data from hard-to-reach populations [29]. The thematic analysis was initiated once all data were collected and had been checked for errors. The transcripts were determined to have reached the level of saturation by the research team. Line-by-line coding was undertaken and developed as a coding tree and assisted by NVivo version 12 to outline the themes. Prospective themes were discussed by the research team before final decisions were made.

Strategies to ensure analytic rigor included having multiple members of the research team review the initial coding and theme categorization at multiple time points during the analysis and using an audit trail to trace how codes and categories evolved throughout the analysis. The summary of the results was also returned to five participants to check the results for accuracy and confirm them [30].

## Results

Parents participating in the study (14 females, and 6 males) included 9 Lahu, 5 Hmong, 1 Lisu, and 5 Akha and ages varied between 18–51 years (35.85 ± 9.69). The healthcare providers (*n* = 20) were predominantly female and the majority were Thai. Community leaders (*n* = 20) who participated in the study ranged in age from 38 to 69 years, with an average age of 51.95 ± 8.43 years. Three parents reported they used the migrant health insurance scheme and two reported that had had no healthcare insurance coverage. Additional demographic information is presented in Table 1.

**Table 1:** Participants’ characteristics

Characteristics	Parents *n* = 20 (%)	Healthcare Providers *n* = 20 (%)	Community Leaders *n* = 20 (%)
Age, years			
Mean **(**SD**)**	35.85 (9.69)	36.85 (7.84)	51.95 (8.43)
Range **(**year**)**	18–51	25–56	38–69
Sex			
Female	14 (70%)	17 (85%)	13 (65%)
Male	6 (30%)	3 (15%)	7 (35%)
Marital status			
Single	0 (0%)	14 (70%)	5 (25%)
Married	15 (75%)	4 (20%)	13 (65%)
Divorced**/**widow**/**separate	5 (25%)	2 (10%)	2 (10%)
Education			
Never attended	7 (35%)	0 (0%)	0 (0%)
Primary school	7 (35%)	0 (0%)	6 (30%)
Secondary school	6 (30%)	4 (20%)	11 (55%)
College/University degree	0 (0%)	16 (80%)	3 (15%)
Ethnicities			
Akha	5 (25%)	2 (10%)	4 (20%)
Lahu	9 (45%)	2 (10%)	4 (20%)
Hmong	5 (25%)	0 (0%)	2 (10%)
Lisu	1 (5%)	0 (0%)	10 (50%)
Thai	0 (0%)	16 (80%)	0 (0%)
Family incomes (Baht)			
< 5,000	19 (95%)	0 (0%)	4 (20%)
5,001–10,000	1 (5%)	4 (20%)	7 (35%)
10,001–15,000	0 (0%)	2 (10%)	8 (40%)
> 15,000	0 (0%)	14 (70%)	1 (5%)
1 Baht = $US 0.028			
Healthcare scheme			
Civil servant medical benefit scheme	0 (0%)	17 (85%)	0 (0%)
Social security scheme	0 (0%)	3 (15%)	0 (0%)
Universal coverage scheme	15 (75%)	0 (0%)	19 (95%)
migrant health insurance scheme	3 (15%)	0 (0%)	0 (0%)
No healthcare insurance	2 (10%)	0 (0%)	1 (5%)

### “Barriers to access”

Barriers to healthcare utilization was the central theme identified when exploring the experiences and perceptions of the hill tribe parents (PR), healthcare providers (HP), and other community leaders (CL) related to healthcare utilization for hill tribe children in the remote areas we studied. Overall, seven sub-themes emerged from the interviews as followed:

### Distance matters

Many participants across the three different sets of participants considered the location of healthcare settings as the principal barrier. As the hill tribe community largely lives in remote, underserved, and difficult-to-access areas, multiple challenges restrict parents’ ability to travel to the nearest town to access care for their children. Specific obstacles mentioned included distance, poor road conditions, travel costs, and safety concerns.

“This is a serious problem for the hill tribe people who live in the mountaintop areas, and public transportation comes only two times a day. Some families do not have a car, and they have to pay 300 baht (US$10), which is the minimum daily wage, to go to the hospital…This means they lose money when visiting the hospital.” (CL4).

“We fully understand that hill tribe people prefer to visit the hospital when their children have health problems; however, they live far from the village and we, as the healthcare providers, cannot visit them either, not only because of the distance but also due to safety concerns.” (HP3).

“We have tried to visit their homes but some of them live in the ‘red’ areas, and drug trafficking is a problem along the Thai–Myanmar border.” (HP1).

“We live so far away, and it is hard to visit the hospital.” (PR5).

### Education and socioeconomic deprivation

Many hill tribe parents mentioned that living in poverty is one of the barriers to accessing healthcare services. Poverty is of course linked to education level which has an impact on parents’ knowledge and ability to care for their children.

“I am poor and have low education, so it is hard for me to understand what the nurse is telling me…and I believe that I did not receive information appropriate to my educational background…For example, vaccination, I did not know what it was and why my child needed it…I just followed what the nurse said and recommended.”(PR12).

Moreover, some healthcare providers addressed the issues that some parents are unable to understand the information provided due to low level of literacy.

The most difficult part of working with hill tribe parents is providing health education; they speak in different languages, and some of them did not attend school. So, when we tell them something and check them…to ensure they have understood they do not give me an answer, they just nod their head.” (HP4).

“Vicious circle…poverty, disease, illness…still exists.” (HP 19).

### Lack of cultural sensitivity

Both parents and community leaders reported that healthcare providers’ behaviors were an important barrier to accessing healthcare services. Some of the parents stated that some healthcare providers were verbally abusive when instructing them on how to care for their child and sometimes healthcare providers did not pay sufficient attention to parents’ concerns and behaved badly towards the parents.

“We were scolded and blamed for not caring for the child…Everything I did seem to be wrong…and they (healthcare providers) did not understand what we were saying or trying to explain.” (PR7).

“Even though we are all human, they should understand that we are not the same, we are different…and we have to do the best for each other.” (PR9).

“Ninety-nine percent of healthcare providers are general Thai…and we are tribal people…we would say that we are different. If it is possible, we would like to have a Hmong nurse who truly understands the Hmong language and truly wants to care for Hmong people in this area.” (CL1).

### Communication problems

The participants in this study reported two major communication problems when communicating with hill tribe population who live in underserve areas. These problems were language barriers which had a negative impact on ability to understand the information and treatment related to the child’s disease. Another problem was complained by many participants in our study about the problem of inadequate mobile phone signal strength or poor internet access to health information.

“The majority of hill tribe parents are illiterate so they cannot read and write but can speak…When they visit the hospital and have a request and if the nurse speaks in complex dialog that they do not understand…they may leave without asking for clarification…it might be because it is too hard to understand.”(CL6).

“Akha is a spoken language with no traditional written language…so I need simple information that helps me understand how to care for my child…Pictures or signs might be helpful if they do not want to explain.” (PR14).

“When you live far from the city, gathering information is very difficult…In the past, we used local broadcasting to keep the people in mountainous areas informed, but now we mostly use mobile devices to send information…It is good in some locations, for my area the signal is very bad.” (CL5).

“When I read a research article about health technology…I felt it was not for the people who live in the mountains and are illiterate.” (HP3).

### Tradition, beliefs, and differences in cultural practice

Believing in faith healers was reported as common practice among the hill tribe parents. When a child becomes sick, parents stated that praying to ancestral ghosts could heal the child rather than visiting the hospital. These preferences offer important insight into why the parents care for their children at home.

“I believe in ancestral ghosts, who protect their descendants from illnesses or death… especially for the children under one year… they are weak and need more protection from our ancestors…visiting a healthcare institution would break the ghosts of our ancestors’ rules.” (PR 8).

“My son had a high fever…we brought him to the hospital and he did not get better…I asked the shaman to come to pray for him…when the shaman came…prayed and apologized for our bad habits…Then he got better; he had no fever…he could eat and play as normal.” (PR13).

Some parents reported that they preferred to use alternative medicine or herbs at home before seeking assistance from healthcare services. The parents attempted to use home remedies instead of visiting healthcare facilities due to their perception that the disease was not severe.

“I felt that I did not need to go to the hospital, I used ‘Hom^1^’ and also encouraged my baby to drink ‘Ya-Keaw^2^’ to reduce the fever.” (PR 16).

“My husband told me that my child does not need the doctor…he uses ‘Hom^1^’ to relieve the fever as he had learned from his parents…I asked… ‘will it work?’ He said… ‘of course yes, my child will get better and survive like him.” (PR2).

^1^Hom,*Strobilanthes cusia,* is Thai-Chinese traditional medical plant has long used in Northern Thailand as anti-pyretic drug.

^2^Ya-Keaw is Thai traditional medicine composed of many herbs and used as anti-pyretic, anti-virus infection, and anti-inflammations.

### Lack of child healthcare professionals

Overall, parents were concerned about their children’s health status and well-being as well as disease prevention, which is provided and monitored by healthcare professionals. However, some parents felt that healthcare providers were novices, lacked specific skills related to the care for the child, and were not well-trained in their field.

“There is no doctor, just a new nurse who was allocated to work here. When my child had a fever, she only gave paracetamol and some cough relief medicine…Later, my child developed pneumonia…he had blue lips and face…and was admitted to the community hospital; that was the last time my child visited the sub-district hospital.” (PR 16).

“My village is far away, and no doctors or nurses want to work here; recently, a nurse, who had worked here for many years, was very kind and good at child health but moved on to work in the city…It is understandable, but it is very sad for us.” (PR2).

“Only one nurse and one public health officer have been working for 3,000 people, and we have no time to train ourselves in child health…once we assessed that they needed more treatment, we then referred the children to the community hospital.” (HP 15).

### Bureaucratic hurdles

Bureaucratic hurdles were identified as a deterrent to utilizing healthcare services. The parents reported that they faced many obstacles to accessing healthcare due to their sometimes-undocumented citizenship status. Access to the Thai health care system requires documents such as proof of health insurance or a referral letter.

“My wife and I are Hmong; we are hill tribe people and migrated from Lao to Thailand 10 years ago. We do not have Thai citizenship, so we used the migrant health insurance scheme when visiting the hospital, while my child is under the UC scheme. However, it was not easy for us to access the hospital…there were time constraints.” (PR 17).

“When my child was sick…and needed to visit the hospital…we did not need to pay but we had to spend one day waiting in the hospital…waiting for everything.”(PR 18).

“Our team has a lot of paperwork to handle as well as caring for our people, so we were unable to do our best with these two tasks at the time.” (HP 12).

## Discussion

This study focuses on the perceptions and experiences of parents, healthcare providers, and community leaders related to healthcare services utilization for hill tribe children between the ages of 0- 5 years. Our study confirms the findings of the limited existing literature that long distances, socioeconomic status, education, healthcare provider-related barriers, and healthcare system structure were the common problems preventing the hill tribe community from receiving or accessing proper health information and services. Consistent with a few prior studies on this population in Thailand residency in underserved and poverty areas results in the need for a full day to visit the hospital [13, 18–20]. Along with other international studies, the study found tribal people faced health disadvantages that resulted from insufficient health services targeted for their specific needs [16, 31, 32]. Further, some healthcare providers also perceived safety problems related to provide home visit program especially in high-drug trafficking areas which represent an obstacle to home healthcare utilization.

Communication problems, including language differences, were identified as an additional barrier to accessing healthcare services. Many parents who participated in this study reported that the health information provided was inadequate in terms of quantity, content, language, and delivery method. The parents also reported that a limited understanding of health conditions and treatments affected their ability to access healthcare services and delivery. This finding is consistent with prior studies on the hill-tribe community [23, 33, 34], which demonstrated that the lack of effectiveness of communication should be addressed by healthcare providers to enhance the hill tribe community’s understanding of, and access to, healthcare services. A similar situation was reported in rural Latin America [35], Albania [36], and Kenya [37]: members of tribal communities refused to vaccinate their children due to not understanding the purpose of vaccines in preventing severe illness in children; this barrier can be addressed through more effective communication and information dissemination strategies [38, 39].

The most common reason for not seeking healthcare given by participants was tradition, beliefs, and cultures. Some participants reported that they believed that their ancestors have a full superpower to protect the child from bad luck and illness at home so they decided to keep the child at home and invited faith healers or shaman to pray at their home. Previous studies conducted on hill tribe populations also support our finding that deep-rooted personal beliefs make it difficult for healthcare providers to enable members of the hill tribe community to visit the hospital [15, 34, 40]. Therefore, health promotion campaigns should involve faith healers to integrate beliefs and traditional health practices for children with modern medical treatment as well as establish home visit program to ensure that parents understand treatments and value healthcare services [20, 41–43].

This study also found that insensitivity of healthcare providers creates barriers to service access. Some parent participants reported that healthcare providers were verbally abusive to them as they provided information. This lack of professionalism and cultural sensitivity undermine patient dignity, reduces trust, and discourages patients from using healthcare services [15, 34, 39]. In this study the majority of the parents considered themselves as low education, underserved, and different from the general Thai population [8, 15]. Thus, according to the vulnerable condition, healthcare providers must strive to provide appropriate and humanized care and look to build trust with the parents, which may improve healthcare service utilization and result in better child health outcomes [11, 15, 33].

Limited availability of healthcare providers, such as pediatric physicians and pediatric nurses, was another significant barrier to utilizing healthcare services [4, 5, 8]. Except for vaccinations, child health services, such as early intervention, oral care, and specific treatments, are largely only available in urban areas or private sectors. These limitations are influenced by the inadequate distribution of healthcare providers in underserved areas, which leads many parents to decide against accessing healthcare services [44, 45]. Inadequate healthcare access is also exacerbated by bureaucratic hurdles, which result in cumbersome paperwork, long waiting times, and complicated appointment processes. Other problems included the stateless people who did not attract an explicit subsidy from the government that can leave hill tribe children even further from accessing healthcare services [16, 46].

### Limitations

The findings of this study should be interpreted with caution due to the small sample size from two districts of remote hill tribe communities which may not represent the views of wider ethnic tribal populations and purposive sampling that could result in study bias. In addition, the majority of the parents who took part in this study had attended primary and secondary school, which is not representative of the education level across the entire hill tribe population; hence, the findings may not be generalizable to other ethnic populations. Moreover, this was a challenging topic to explore in a single interview, which may have limited the depth of the discussions.

## Conclusions

In order to achieve health equity goals for Thailand, the government must increase access to and quality of healthcare services for hill tribe children who are especially vulnerable to a wide range of health issues. This study revealed several important barriers to access to healthcare services for hill tribe children, including geographical and communication challenges, lack of knowledge and information, healthcare perceptions and beliefs, and unsupportive healthcare environments. In addition, the study findings suggest that hill tribe parents, particular those with lower socioeconomic status, need to improve their health literacy. Setting higher standards for training and professionalization of health care workers who provide health care and education to hill tribe children and their parents is an initial step.

## Supplementary Information

**Additional file 1:** Semi-structured interviewguide.

## Data Availability

The audio recordings and transcripts analyzed in this study are not publicity available as it is possible to sufficient deidentify them to maintain participant privacy and confidentiality. Addition deidentify information can be obtained from the corresponding author upon reasonable request.

## Abbreviations

EPI: Expanded Program on Immunization
FGD: Focus group discussion
SDGs: Sustainable Development Goals
UC: Universal Coverage Healthcare Scheme
UN: The United Nations
