# Supplementary material for: Healthcare service utilization of hill tribe children in underserved communities in thailand: Barriers to access
Source: BMC Health Serv Res. 2022 Sep 2;22:1114. doi: 10.1186/s12913-022-08494-1 (PMC9438234; doi:10.1186/s12913-022-08494-1)
Supplement: Supplementary file 1 — Additional file 1: Semi-structured interviewguide. [file 12913_2022_8494_MOESM1_ESM.docx]

**Semi-structured interview guide**

**(Parents).**

1. ‘Could you please tell me about the experience of caring for the child when they was young especially age under 5 years?”,

‘Could you explain more.

1. “Did your experience any disappointed situation when visiting the hospital?”,

‘Can you share with me how you dealt with the situations?

1. ‘“Has your child(ren) missed or delayed a vaccine?”.

‘Could you tell me more’

1. ‘What are the challenges that you faced when visiting the hospital when your child sick?’

‘What kind of support have you had when visit the hospital?’

1. ‘How does your spouse/ grandparents/ child’s sibling/ friends/neighbors/ /communities support you in caring for the child ?’

“how about your beliefs ?”

1. ‘Can you tell me what are yours expectation for child health services?’

‘If you have a chance to talk with the stakeholders, what would you like to tell them about caring for hill tribe children?’

**Semi-structured interview guide**

**(Healthcare providers and Community leaders).**

- - - 1. ‘Could you please tell me about the experience of caring for the hill tribe child especially age under 5 years?”,

‘Could you explain more.

1. “Please tell me more about the barriers when providing care for hill tribe children?”,

‘Can you share with me how you dealt with the situations?

‘Could you tell me more’

1. ‘What are the challenges that you faced when providing care for hill tribe children at the hospital and community?’
2. ‘What kind of support have you had from the government or community?’
3. “What do you think about the current healthcare services for hill tribe children delivered by community leaders or VHVs?”

‘Can you tell me what are yours expectation for child health services?’

1. “What possible goals for caring the hill tribe children that do you want to achieve?”
2. ‘If you have a chance to talk with the stakeholders, what would you like to tell them about caring for hill tribe children?’
